# The ATRXt Protein Represses rDNA Transcription While Mirroring ATRX Interactions and Heterochromatin Localization

**DOI:** 10.3390/ijms27073103

**Published:** 2026-03-29

**Authors:** Mathieu G. Levesque, David J. Picketts

**Affiliations:** 1 Regenerative Medicine Program, Ottawa Hospital Research Institute, Ottawa, ON K1H8L6, Canada; matlevesque@ohri.ca; 2 Departments of Biochemistry, Microbiology, & Immunology, University of Ottawa, Ottawa, ON K1H8M5, Canada; 3 Department of Cellular and Molecular Medicine, University of Ottawa, Ottawa, ON K1H8M5, Canada; 4 Departments of Medicine, University of Ottawa, Ottawa, ON K1H8M5, Canada

**Keywords:** chromatin remodeling, alternative splicing, ribosome biogenesis, heterochromatin, nucleolus

## Abstract

The *ATRX* gene encodes an SWI/SNF-type chromatin remodeling protein that is critical for proper development of the mammalian central nervous system and musculoskeletal system. While significant progress has been made in understanding the molecular functions of the full-length (FL) ATRX protein, there is still very little known about its conserved alternative spliceoform, ATRXt. ATRXt is a truncated isoform of ATRX which lacks the entire SWI/SNF domain due to the retention of intron 10, which results in the in-frame addition of 61 unique amino acids (exon 10a) at its C-terminus. Here, we demonstrate that ATRXt accounts for 5–20% of total ATRX protein levels, while showing tissue- and differentiation-specific changes in expression levels compared to its full-length counterpart. ATRXt shows enriched localization at H3K9me3-positive heterochromatin but not at PML-nuclear bodies, while physically interacting with the known FL-ATRX protein partners, DAXX and HP1α. Exon 10a can target a GFP-fusion protein to the nucleolus, but removal of exon 10a from ATRXt does not prevent nucleolar localization. Finally, re-introducing ATRXt into the ATRX-negative U2OS cell line reduced rRNA transcription and severely hampered cell growth, similar to previous studies using FL-ATRX. Our study highlights that ATRXt has many of the same properties as FL-ATRX, suggesting that some roles of ATRX do not require remodeling activity, while highlighting the need to distinguish ATRXt’s functions from those of the full-length protein.

## 1. Introduction

Alpha-thalassemia intellectual disability syndrome X-linked (ATRX) is a unique SWI/SNF-like chromatin remodeling protein. ATRX has an ATPase/helicase domain characteristic of this family but is considered an “orphan” chromatin remodeler due to its distinctive protein domains outside of its C-terminally located SWI/SNF domain [1,2]. It is now well-established that ATRX acts on heterochromatin. The protein can directly bind H3K9me3, a mark of constitutive heterochromatin, via its N-terminally located ATRX-DNMT3A-DNMT3L (ADD) domain [3]. Stable interactions with HP1α, EZH2, and MeCP2, all heterochromatin-associated proteins, have been reported and further support a role for ATRX at these regions [4,5,6,7,8,9]. The best characterized interaction is with DAXX, a histone chaperone. The ATRX-DAXX complex functions to deposit histone H3.3 at repetitive genomic sequences with a propensity to form G4 quadruplexes, most notably telomeric and pericentromeric heterochromatin regions [10,11]. It is in these regions that ATRX is thought to be crucial for maintaining genomic stability, especially in the context of DNA replication. The role of ATRX in telomere stability is particularly notable in the context of the Alternative Lengthening of Telomeres (ALT) cancer phenotype. Instead of upregulating telomerase, ALT cancers maintain their telomeres by a homologous recombination-based mechanism [12]. ATRX loss in cancer is highly correlated with the occurrence of ALT, and reintroduction of the protein in ALT+ cancer cells can suppress the ALT phenotype [13,14,15,16]. In non-cancer cells, loss of ATRX results in upregulation of TERRA transcription and replicative damage at telomeres [11,17]. Replicative damage is thought to occur upon loss of ATRX due to the accumulation of G4 secondary structures, which can stall the replication fork and lead to genomic instability [18]. ATRX is proposed to interact with the MRE11 exonuclease, preventing its degradation of stalled replication forks [19]. More recently, ATRX was shown to be capable of regulating euchromatic regions by altering gene expression, chromatin accessibility, and transcription factor binding [20].

ATRX mutations in the germline result in the ATR-X syndrome, a rare and severe neurodevelopmental disorder [21]. Pathogenic ATRX variants are highly enriched within the ADD and SWI/SNF domains. Over 192 variants have been cataloged and many are predicted to be functional hypomorphs, with some data suggesting that complete loss of ATRX function is non-viable [22]. Indeed, early ablation of *ATRX* in mice is embryonic lethal, while conditional knockout models of *ATRX* have highlighted a role in mediating replicative stress damage in proliferating neural progenitor and skeletal muscle cells [19,23,24,25]. More recent studies have also suggested a role for ATRX at rDNA. ChIP-seq studies show ATRX directly binding to rDNA [26]. Loss of ATRX in mouse embryonic stem cells (mESCs) was associated with a 50% reduction in rDNA copy number, and ATRX-mutated cancers consistently have a reduction in rDNA copy numbers versus ATRX intact tumors [27]. Proteomics data suggests that ATRX associates with several proteins implicated in ribosome biogenesis, including FAM207A and IMP321 [28].

Despite significant progress in studying the biochemical function of ATRX, the ATRX locus gives rise to two major transcripts; one encodes the full-length protein (FL-ATRX), and the other is a truncated isoform called ATRXt. The ATRXt isoform is observed in both murine and human tissues, but little is known about its specific biochemical functions, and most studies simply ignore its role altogether [4,29,30]. ATRXt is generated by the inclusion of intron 10 of the full-length transcript, which remains in-frame and adds 61 amino acids after exon 10 (hereby referred to as exon 10a), followed by an in-frame stop codon and a downstream polyadenylation signal. There is a high degree of conservation of these 61 amino acids between mice and humans; however, their functional significance has not been determined [30]. The ATRXt spliceoform encodes a protein that retains the ADD domain but lacks the SWI/SNF domain of FL-ATRX, thereby presumably eliminating nucleosome remodeling activity. Available biochemical data indicates that ATRXt can localize at pericentromeric heterochromatin, colocalizing with HP1β in mouse embryonic fibroblasts (MEFs) [30]. ATRXt transcript is expressed ubiquitously and at different levels across various human tissues [30]. It was also shown via co-IP that a proportion of FL-ATRX and ATRXt may exist in a complex [30]. Taken together, existing data is scarce, albeit suggestive of an important and largely unexplored biological role for ATRXt.

We report here an initial characterization of the biochemical functions of ATRXt. We confirm that the protein can interact directly with several known FL-ATRX protein partners, including DAXX. We show that there is an enrichment of ATRXt in the nucleolus, where it is recruited in an RNA-dependent manner. Importantly, we show that ATRXt can repress rRNA transcription, demonstrating an ATP-independent function for ATRX in gene expression regulation and revealing a previously unknown function for ATRXt. Overall, our findings introduce the molecular function of ATRXt and demonstrate significant involvement for the isoform in the regulation of ribosome biogenesis.

## 2. Results

### 2.1. The Amino Acid Sequence and Expression Profile of ATRXt Compared to FL-ATRX

**Figure 1:**
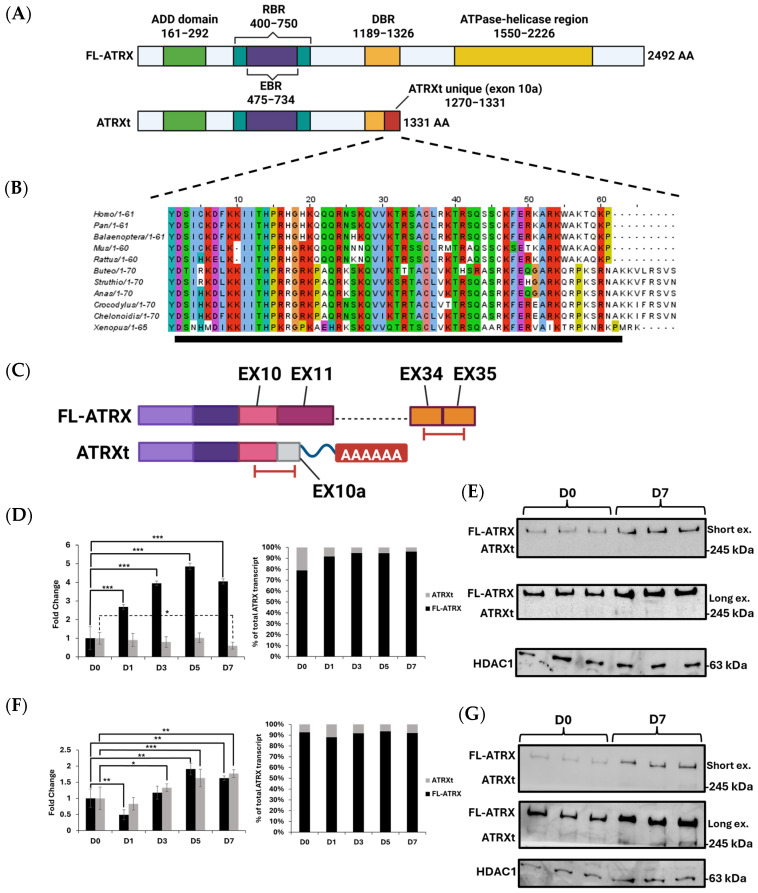
ATRXt structure and expression suggest an important biological function. (**A**) Protein domains of FL-ATRX and ATRXt. ATRXt retains the exact same amino acid sequence as FL-ATRX except for its C-terminal 61 amino acids, dubbed exon 10a, which are uniquely expressed in ATRXt. EBR, EZH2-binding region; RBR, RNA-binding region; DBR, DAXX-binding region. (**B**) Clustal Omega (version 1.2.4) alignment of ATRXt exon 10a amino acid sequence, showing homology in mammals, birds, reptiles and amphibians. Amino acids were color-coded according to Clustal-X [31]. The black bar indicates the amino acid sequence predicted to be a nucleolar localization sequence [32]. (**C**) Schematic outlining alternative splicing of ATRX. The ATRXt isoform arises due to read through of exon 10 into FL-ATRX intron 10, where ATRXt exon 10a is encoded. Target regions for amplification for detection by qPCR are denoted by the red bars. (**D**) Left, the levels of ATRXt and FL-ATRX mRNA measured in differentiating LUHMES cells, comparing expression in undifferentiated cells (D0) and after seven days of differentiation (D7). Right, the proportion of ATRX transcript made up by FL-ATRX and ATRXt at the same timepoints as in the graph on the left. (**E**) Western blot showing ATRX expression in LUHMES cells in undifferentiated (D0) cells and after seven days of differentiation (D7). Short and long exposures are shown. The primary antibody used to detect ATRX is 39F, which targets amino acids 85–319 of ATRX. (**F**) The same as in (**D**), measured in differentiating SH-SY5Y cells. (**G**) The same as in (**E**), measured in SH-SY5Y cells *, *p* < 0.05, **, *p* < 0.01, ***, *p* < 0.001.

The ATRXt cDNA encodes a protein of 1331 amino acids, a size reduction of 47% compared to the 2492-amino-acid protein encoded by FL-ATRX (Figure 1A). An examination of the amino acid sequence of ATRXt reveals that it retains the ADD domain, the RNA-binding region, and a significant portion of the DAXX-binding region (Figure 1A). Crucially, the ATPase-helicase domain (amino acids 1550–2226) of FL-ATRX is not present in the ATRXt isoform, being replaced by a unique sequence of 61 amino acids encoded within exon 10a (Figure 1A). Using Clustal Omega (version 1.2.4), we examined the amino acid homology of the ATRXt-unique exon 10a across several different animal species and observed a high degree of conservation of the sequence (Figure 1B). Using a computational prediction tool, we were able to identify exon 10a as containing a putative nucleolar localization signal, potentially explaining the strong conservation of this exon (Figure 1B) [30]. We also examined the homology of the genomic sequence corresponding to exon 10a. We noted that the species conservation of the genomic sequence decreased immediately after the coding sequence of exon 10a (Figure S1B). Comparing the genomic sequence encoding exon 10a to that of an equivalent intronic sequence, namely the 200 intronic nucleotides immediately downstream of FL-ATRX exon 12, showed that the species conservation of exon 10a was greater than 2-fold higher than that of the first 200 nucleotides of intron 12 (Figure S1B). These findings indicate a selective pressure to maintain the sequence of exon 10a, which may be suggestive of an important biological function.

FL-ATRX protein is known to be a crucial regulator of cellular differentiation in the context of both neural tissue and skeletal muscle [23,24,25]. As such, we next compared FL-ATRX and ATRXt expression dynamics within two different neuronal cell lines under both growth and differentiating conditions. We initially focused on the human cell lines, SH-SY5Y and LUHMES, which are immortalized cell lines derived from neuroblastoma tumor and human midbrain neuronal precursor cells, respectively. C2C12 mouse myoblast cells were used to compare expression differences during muscle cell differentiation. Using RT-qPCR, we measured the levels of FL-ATRX and ATRXt transcripts throughout a seven-day differentiation time course for each cell line (Figure 1C–G and Figure S1A,C). We found that ATRXt comprised 5–20% of the total ATRX transcript, depending on the cell line and the timepoint of differentiation (Figure 1D,E and Figure S1A).

In the LUHMES cell line, the proportion of ATRXt transcript decreased from 20% of total ATRX transcript in the proliferating cell population to 5% after 7 days of differentiation. The overall mRNA levels of ATRXt remained constant, showing only a marginal decrease after 7 days of differentiation. The regulation of FL-ATRX mRNA was quite different, with mRNA levels increasing 5-fold over the differentiation timecourse (Figure 1D). Similarly, immunoblots of each ATRX isoform were consistent with the RT-qPCR data, with FL-ATRX protein levels increased after 7 days of differentiation and ATRXt protein levels decreased (Figure 1E). In contrast to the LUHMES cells, SH-SY5Y cells showed a modest increase in FL-ATRX and ATRXt, both at the mRNA and protein levels (Figure 1F,G). Indeed, all three cell lines examined showed similar proportions of ATRXt to FL-ATRX mRNA in proliferating cells and different responses when induced to differentiate. During the C2C12 muscle cell differentiation timecourse, both isoforms decreased (Figure S1A). Taken together, these data suggest that the ATRXt isoform undergoes both tissue-specific and differentiation-specific regulation, which can differ from the regulation of the FL-ATRX isoform.

### 2.2. ATRXt Has a Subnuclear Localization Distinct from FL-ATRX

**Figure 2:**
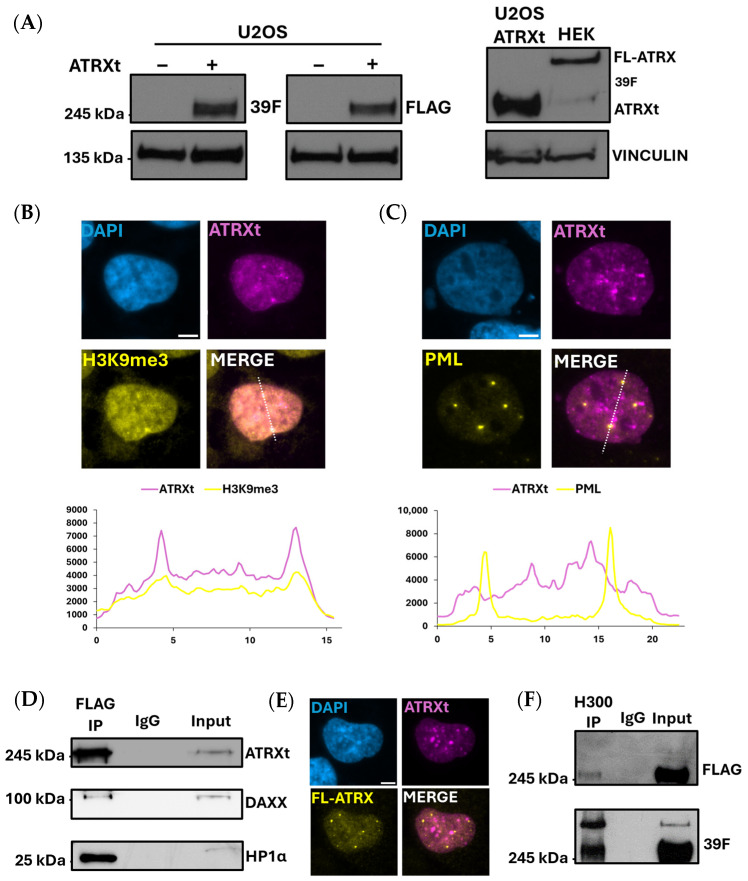
ATRXt subnuclear localization and partner proteins. (**A**) Left, Western blots of U2OS cell lysate after transfection of ATRXt, detected by both a FLAG antibody and an N-terminal ATRX antibody (39F); right, Western blot of U2OS cell lysate after transfection of ATRXt compared to endogenous ATRXt and FL-ATRX detected in HEK 293 cell lysate. (**B**) Co-staining of overexpressed ATRXt in U2OS cells with H3K9me3. Dotted lines indicate segments used for fluorescence intensity measurements of each channel, shown below. (**C**) Same as in (**B**), showing co-staining of ATRXt with PML. (**D**) Western blot for FLAG, DAXX and HP1α following FLAG-IP of ATRXt from transfected U2OS cells. (**E**) Co-localization of ATRXt and FL-ATRX after co-transfection in U2OS cells. (**F**) Western blot probed with an antibody specific to ATRXt (FLAG, top) and an antibody not specific to either FL-ATRX or ATRXt (39F, bottom) following IP of FL-ATRX from U2OS cells co-transfected with FL-ATRX and ATRXt.

To interrogate ATRXt’s function, we made use of the U2OS cell line, which has been shown to harbor a genomic deletion of the ATRX locus and to lack any ATRX expression, and has proven useful as a model to characterize FL-ATRX function following exogenous expression [14,16]. We began by generating an ATRXt cDNA construct with a 3XFLAG tag at the N-terminus (Figure 2A). Transient transfection of U2OS cells with FLAG-ATRXt showed that the protein expressed was of a similar size to endogenous ATRXt produced in HEK 293 cells and was easily detectable with both ATRX-specific (39F) and FLAG antibodies (Figure 2A). To examine the subnuclear distribution of ATRXt, we performed immunofluorescence (IF) microscopy using a FLAG antibody on transfected U2OS cells. This experiment revealed that FLAG-ATRXt localizes to regions of heterochromatin as denoted by line plots showing co-localization with H3K9me3 antibody staining (Figure 2B). FLAG-ATRXt also co-localized with EZH2, H3K27me3 and HP1α (Figure S2A–C). In contrast, we did not observe co-localization of ATRXt with PML, a protein previously shown to co-localize with FL-ATRX in multiple cell lines (Figure 2C) [10]. As expected, FL-ATRX showed colocalization with both H3K9me3 and PML when overexpressed in U2OS cells (Figure S2D,E).

Examination of direct protein–protein interactions by co-immunoprecipitation (co-IP) of ATRXt from transfected U2OS cells showed that it interacts with HP1α, a heterochromatin factor which is known to bind ATRX via an N-terminal domain (Figure 2D). ATRXt partially retains the primary DAXX-interacting domain of FL-ATRX, which is amino acids 1189–1326 [33]; the two ATRX isoforms share the same amino acid sequence up to amino acid 1270 (Figure 1A). Co-IP of ATRXt with DAXX showed that despite retaining only 59% of the DAXX-interacting domain, ATRXt can still interact with DAXX (Figure 2D). To more accurately assess the difference in nuclear distribution between FL-ATRX and ATRXt, we conducted co-transfections of FL-ATRX and ATRXt into U2OS cells. Analysis by IF showed no obvious colocalization, with a clear number of distinct foci unique to one isoform or the other (Figure 2E). Nonetheless, co-IP of co-transfected cells with a C-terminal ATRX antibody (H300) that is specific to FL-ATRX demonstrated an association with ATRXt, suggesting that the two isoforms may be able to form a dimer in vivo within the nucleoplasm (Figure 2F). Our data confirm that ATRXt retains protein interactions with known FL-ATRX interactors (DAXX, HP1α) and demonstrated localization at H3K9me3-enriched heterochromatin. However, the ATRXt protein does show differences in subnuclear localization suggestive of unique functions for the truncated isoform.

### 2.3. ATRXt Localizes to the Nucleolus in an RNA-Dependent Manner

**Figure 3:**
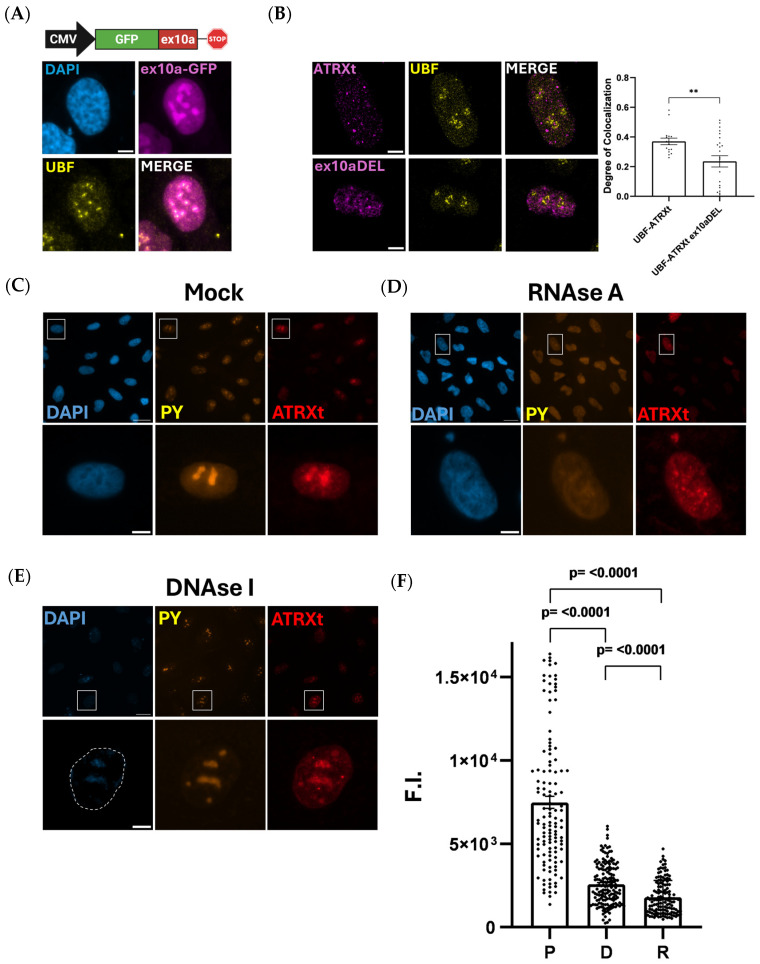
ATRXt localizes to the nucleolus in an RNA-dependent manner. (**A**) Top, schematic of construct used to express GFP fused to ATRXt exon10a. Below, co-staining of exogenously expressed exon10a-GFP with UBF in U2OS cells. (**B**) Immunofluorescence showing colocalization of WT ATRXt (top) and ATRXt with exon10a deleted (bottom) with UBF. Right, the degree of colocalization (Manders’ coefficient) of either protein with UBF. **, *p* < 0.01. Number of nuclei counted per condition: UBF-ATRXt = 16, UBF-ATRXt ex10aDEL = 23. (**C**) U2OS^ATRXt^ cells were either permeabilized or treated with (**D**) RNAse A (200 μg/mL) or (**E**) DNAse I (100 μg/mL) 24 h after induction of ATRXt expression. (**F**) Quantification of ATRXt nucleolar intensity after permeabilization (P), DNAse I (D) or RNAse A (R) treatment. The square on the top panels denotes the zoomed in area shown on the bottom panels. The dashed area in (**E**) encircles the nuclear area. F.I. = fluorescence intensity (arbitrary units). Number of nucleoli counted per condition: P = 124, D = 161, R = 136. Average Number of nucleoli per cell = 4.01, *n* = 136. Scale bar = 20 μm, inset = 5 μm.

An in silico examination of the exon 10a amino acid sequence for potential functional domains identified a putative nucleolar localization sequence (NoLS; Figure 1B). To test the functionality of the NoLS, we generated a fusion protein in which exon 10a was inserted in-frame to a GFP expression cassette (ex10a-GFP). Transfection of ex10a-GFP into U2OS cells demonstrated a pattern consistent with nucleolar localization, which was confirmed by co-staining with the nucleolar protein UBF (Figure 3A and Figure S3A). GFP expressed on its own shows no visible nucleolar enrichment (Figure S3B). To further probe the requirement for exon 10a to direct ATRXt to the nucleolus, we generated a C-terminally GFP-tagged ATRXt construct with exon 10a deleted (ex10a DEL). We compared co-localization of the ATRXt WT (containing a 3XFLAG tag) and the GFP-ATRXt ex10a DEL construct after co-transfection into U2OS cells. We found that co-localization of ATRXt with UBF was significantly reduced after deletion of exon 10a (Figure 3B). In addition, we observed that ATRXt ex10a DEL was still able to localize to H3K9me3 heterochromatin (Figure S3B). Thus, while exon 10a promotes ATRXt nucleolar localization, it is not essential nor does it appear to be crucial for ATRXt targeting to heterochromatin.

Previous studies highlighted the importance of RNA for FL-ATRX tethering to heterochromatin, as treating cells with RNAse A significantly impacts the localization of FL-ATRX [8]. Importantly, the putative RNA-binding domain of FL-ATRX was identified between amino acids 450 and 700, which is retained in ATRXt [8]. We reasoned that since FL-ATRX is known to bind both DNA and RNA [8,34], implementing our own nuclease treatments targeting either DNA or RNA would inform us on how nucleic acids regulate the subnuclear distribution of ATRXt. To this end, we generated a U2OS cell line with stably integrated ATRXt under the control of a doxycycline promoter (hereby referred to as U2OS^ATRXt^). After confirmation of doxycycline inducible expression of ATRXt (Figure S3C), cells were induced for 24 h then treated with either DNAse I or RNAse A for 20 min prior to fixation and IF-staining. We compared these cells with those that were doxycycline-induced and permeabilized but not treated with nucleases (mock treated). IF staining revealed an enrichment of ATRXt within the nucleolus that was readily apparent after mock or DNAse I treatment of the cells but was not as prevalent and more dispersed in RNAse A-treated cells (Figure 3C–E). To quantify this effect, we calculated the fluorescence intensity of ATRXt in the nucleoli of the cells in all conditions tested. Bright pyronin Y-stained regions were used as a proxy for nucleoli in all conditions except for RNAse A-treated cells, where we stained cells with UBF to denote nucleoli. Pyronin Y is an RNA-intercalating dye, which when used in IF highlights the RNA-rich nucleoli. UBF is a DNA-binding transcription factor critical for RNA polymerase I-mediated transcription, which transcribes the ribosomal RNA. Since UBF is a DNA-binding protein, its nuclear distribution is unaffected by RNAse A treatment of the cells. Quantification confirmed that the ATRXt signal was significantly higher in nucleoli in both mock and DNAse I-treated cells compared to RNAse A-treated cells (Figure 3F). In addition, we generated a U2OS line with stably integrated FL-ATRX under the control of a doxycycline promoter (U2OS^ATRX-FL^) (Figure S3D). We performed the same treatments as described for U2OS^ATRXt^ with these cells and found similar results, wherein FL-ATRX fluorescence intensity was significantly higher in the nucleoli of cells after mock or DNAse I treatment versus RNAse A-treated cells (Figure S4A–D).

**Figure 4:**
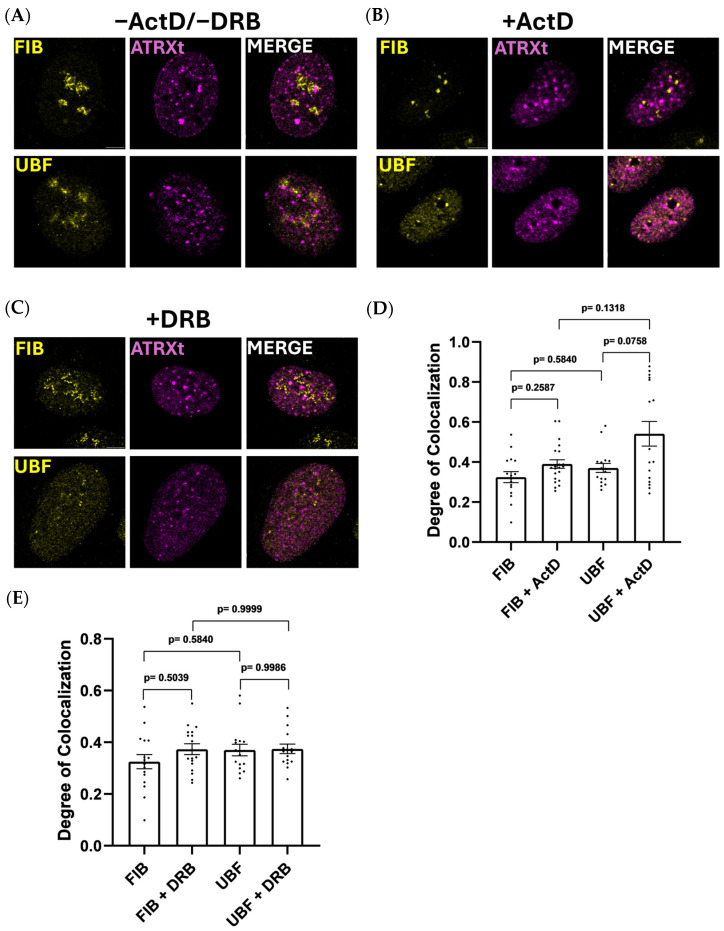
ATRXt colocalization with nucleolar sub-compartments. U2OS^ATRXt^ cells were treated with doxycycline for 24 h and subsequently co-stained for ATRXt with UBF or fibrillarin (FIB), markers of the FC and DFC, respectively. Cells were either untreated (**A**), treated with 0.5 µg/mL Actinomycin D for 2 h (**B**) or treated with 25 µg/mL 5,6-dichloro-β-D-ribofuranosylbenzimadole for 2 h (**C**). (**D**) The degree of colocalization (Manders’ coefficient) of ATRXt with UBF and fibrillarin before and after Actinomycin D treatment. (**E**) The degree of colocalization (Manders’ coefficient) of ATRXt with UBF and fibrillarin before and after 5,6-dichloro-β-D-ribofuranosylbenzimadole treatment. Number of nuclei counted per condition: FIB = 16, UBF = 16, FIB w/ActD = 21, UBF w/ActD = 16, FIB w/DRB = 17, UBF w/DRB = 16.

The nucleolus can be sub-divided into three sub-nucleolar compartments, namely the fibrillar center (FC), the dense fibrillar component (DFC), and the granular component (GC) [35,36]. The rDNA is located in the FC and the 47S pre-rRNA transcription occurs at the FC/DFC interface. Early and late rRNA processing occurs within the DFC and GC, respectively. Previous studies have demonstrated that proteins co-localized within specific sub-compartments remain associated during nucleolar disassembly induced by different treatments, specifically Actinomycin D (ActD) treatment to inhibit RNA polymerase I and arrest pre-RNA transcription, or treatment with 5,6-dichloro-β-D-ribofuranosylbenzimadole (DRB) that inhibits casein kinase 2 to uncouple the rRNA processing machinery from rRNA transcription [37,38,39,40]. Using a similar approach, we reasoned that ActD and DRB treatments could help us to identify which sub-compartment that ATRXt resides within. Following doxycycline induction of ATRXt expression, cells were treated with 0.5 ug/mL ActD or 25 ug/mL DRB for 2 h and then analyzed for ATRXt localization to specific sub-compartments identified by co-localization with UBF (FC marker), fibrillarin (DFC marker) or nucleolin (GC marker). Both before and after ActD/DRB treatments, we observed partial co-localization of ATRXt with fibrillarin and UBF (Figure 4A–C). Partial co-localization with nucleolin was also seen prior to chemical treatments. After the chemical treatments, nucleolin is completely dispersed within the nucleoplasm while ATRXt retains its speckled distribution (Figure S5A–C). We reasoned that it was therefore unlikely that ATRXt was localized to the GC to any significant degree. To quantify the association of ATRXt with the FC and DFC, we measured the degree of colocalization of ATRXt with UBF and fibrillarin, respectively. ATRXt did show a moderate degree of colocalization with UBF and fibrillarin in all conditions tested (Figure 4D,E). While not statistically significant, we observed an upward trend in ATRXt’s association with UBF after ActD treatment (Figure 4D). DRB treatment did not reveal any differences in ATRXt association with either UBF or fibrillarin versus untreated cells (Figure 4E).

The same treatments on U2OS^ATRX-FL^ cells showed similar results (Figures S5A–C and S6A–E). FL-ATRX had a moderate degree of colocalization with UBF and fibrillarin in all conditions tested. While ActD treatments showed no differences in FL-ATRX association with either UBF or fibrillarin, DRB treatment showed significantly more FL-ATRX association with UBF versus fibrillarin (Figure S6D,E).

The enrichment of ATRXt and FL-ATRX in the nucleoli of cells after mock treatment and DNAse I treatment suggests that both isoforms have some enrichment in the nucleolus, where they are insolubly bound, and the proteins’ enrichment is likely dependent on both a DNA and RNA component. While inconclusive as to which component of the nucleolus ATRXt is enriched in, chemical treatments suggest an enrichment in both the FC and DFC, and perhaps especially so in the FC.

### 2.4. ATRXt Is a Negative Regulator of rRNA Transcription

**Figure 5:**
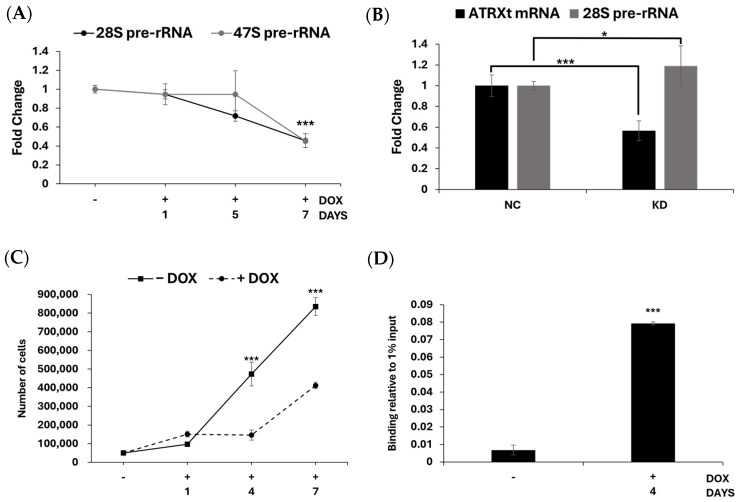
ATRXt expression in U2OS cells represses rRNA levels and impedes cellular proliferation. (**A**) 47S and 28S rRNA levels in U2OS^ATRXt^ at selected timepoints after doxycycline treatment relative to rRNA levels in cells with no doxycycline added. (**B**) ATRXt mRNA and 28S pre-rRNA levels 24 h after siRNA knockdown of ATRXt (KD) in HeLa cells measured relative to HeLa cells treated with non-targeting negative control siRNA (NC). (**C**) Growth of U2OS^ATRXt^ cells at selected timepoints after plating, grown in the presence or absence of doxycycline (+/−DOX). 50,000 cells were plated on the first day. (**D**) ATRXt enrichment at 28S rDNA determined by ChIP-qPCR. ATRXt binding was measured in the presence or absence of doxycycline (+/−DOX), relative to 1% input. *n* = 3 for all experiments, * = *p* < 0.05, *** = *p* < 0.001.

Given the enrichment of ATRXt to the nucleolus, we next asked whether it localized to rDNA and was involved in regulating rRNA output. To test this, we induced ATRXt expression in our U2OS^ATRXt^ cells and measured the level of rRNA at 1, 5, or 7 days later. We found that after 7 days of ATRXt expression, the levels of 28S pre-rRNA were reduced by 50% (Figure 5A). We confirmed the specificity of this effect by measuring rRNA levels in a U2OS cell line which we engineered to express the ZsGreen fluorescent protein (U2OS^ZsGreen^) in a doxycycline-inducible manner, which showed no statistically significant change in 28S rRNA levels after 7 days of ZsGreen expression (Figure S7A). To gauge whether the reduction in 28S transcript levels was because of reduced transcription or an rRNA processing defect, we also measured the level of the 47S pre-rRNA. We found that the 47S pre-rRNA was reduced by a similar amount to the 28S transcript and at the same timepoint, suggesting that the reduction was a transcriptional change rather than a processing defect (Figure 5A). A similar reduction in rRNA was also measured in a U2OS stable cell line that was engineered to constitutively express ATRXt (Figure S7B). As with the 28S rRNA levels, 47S rRNA levels in the U2OS^ZsGreen^ cells remained unchanged after 7 days of ZsGreen expression (Figure S7A).

To test if the reduction in ATRXt levels could also influence rRNA transcription, we designed siRNA oligonucleotides targeting exon 10a to knock down ATRXt expression specifically without affecting FL-ATRX levels. We transfected the exon 10a siRNA oligonucleotides into HeLa cells that express both FL-ATRX and ATRXt. Following transfection, RT-qPCR was used to demonstrate that we achieved a knockdown of ATRXt mRNA by approximately 50%, while confirming that FL-ATRX mRNA levels were unaffected (Figure 5B and Figure S7C). Similarly, we demonstrated that the levels of 28S pre-rRNA increased by approximately 20% in these cells, a modest but statistically significant increase (Figure 5B). As ribosome biogenesis is intimately linked to the metabolic rate of a cell and thus its capability for proliferation [41], we tested if the growth of the U2OS^ATRXt^ cells was affected by ATRXt expression and subsequent reduction in rRNA transcription. The growth of U2OS^ATRXt^ was significantly affected by ATRXt expression, slowing the cells’ growth rate significantly (Figure 5C). Confirming the specificity of this effect to ATRXt expression, the growth rate of the U2OS^ZsGreen^ cells was unaffected by ZsGreen expression (Figure S7D). A significant reduction in rRNA levels and cellular growth was also observed in our U2OS^ATRX-FL^ cells after FL-ATRX expression (Figure S7E,F). Finally, we confirmed direct ATRXt binding to the rDNA locus by performing ChIP-qPCR using primers specific to the 28S rDNA (Figure 5D). Our data thus suggests that ATRXt is a negative regulator of rRNA transcription, which is likely linked to the marked proliferation defect seen in U2OS cells when ATRXt is expressed.

## 3. Discussion

Alternative splicing of transcripts generates an enormous amount of biological complexity in eukaryotes. In humans, nearly all multi exonic genes give rise to at least two alternative spliceoforms, highlighting the importance of this often-overlooked biological process [42,43,44,45]. The biological relevance of alternative spliceoforms extends to chromatin remodeling proteins and their accessory subunits, with their function now beginning to be interrogated [46,47,48,49,50]. Here we investigated the biological role of ATRXt, a short isoform of ATRX that lacks the SWI/SNF-like chromatin remodeling domain. We report that ATRXt retains properties of its full-length counterpart, including the ability to localize to H3K9me3 decorated heterochromatin and to interact with protein partners HP1α and DAXX. While the novel exon 10a encoded a nucleolar localization sequence, it was not required for subcellular localization. We demonstrated that both DNA and RNA were critical for nucleolar targeting of ATRXt where it functions in the repression of rRNA transcription. Given the similar findings observed with FL-ATRX, we conclude that the nucleolar functions of ATRX are independent of chromatin remodeling activity. Moreover, unique functions of ATRXt remain to be defined.

Nonetheless, this study provides important insight into the role that specific domains have on ATRX function. The localization of ATRXt with the constitutive heterochromatin mark H3K9me3 and its interaction with HP1α confirm that the N-terminus is critical for tethering ATRX to heterochromatin, via both the ADD domain (aa 161–292) and the HP1 interaction domain (aa 581–594) [3,4,51]. Importantly, it also demonstrates that chromatin remodeling activity is not required for heterochromatin binding. We also show that while ATRXt localized to the nucleolus, exon 10a was not required, suggesting that the other previously defined NLS sequences (aa 685–697 and 1126–1196) can direct nucleolar targeting [52]. Furthermore, the co-IP of ATRXt with DAXX indicates that this binding domain (aa 1189–1326) can be refined to amino acids 1189–1270.

Interestingly, we were also able to co-immunoprecipitate FL-ATRX with ATRXt, suggesting that a dimerization domain exists within the N-terminal half of the protein, while confirming an earlier report in mouse fibroblasts [30]. The potential for FL-ATRX and ATRXt to form a heterodimer in vivo is tantalizing and would represent a novel ATRX complex to study. Indeed, previous work has hinted at the possibility of ATRX functioning as a dimer. The initial purification of the ATRX-DAXX complex showed that the two proteins co-fractionated in a complex of 1MDa, which is much larger than the additive size of the two proteins (~360kDa) [10]. This discrepancy could be explained by the ATRX-DAXX complex functioning as a tetramer rather than a dimer, which is not unprecedented. The MORC family of ATP-dependent chromatin remodelers is known to form homodimers as a prerequisite for chromatin remodeling [53]. It has also been suggested that SMARCA5, one of the human ISWI ATP-dependent chromatin remodelers, functions as a dimer during nucleosome sliding [54]. More work is needed to explore this possibility and how it might affect functionality, particularly since ATRX shows dosage sensitivity and only the full-length isoform contains remodeling activity [10,55].

It has been known for some time that FL-ATRX can localize to rDNA and may function in maintaining the stability of the repeat region [27,29,56]. ChIP studies have shown that FL-ATRX binds to the coding region of rDNA, with the highest levels occupying the region encoding 28S pre-rRNA [26,27]. Intriguingly, we now demonstrate that ATRXt can similarly localize to the rDNA locus through binding to 28S rDNA (Figure 5D). Our data show that ATRXt can act as a negative regulator of rRNA transcription, proving that ATRX has an impact on gene expression in an ATP-independent manner. Loss of FL-ATRX is linked with loss of repressive heterochromatin, specifically loss of H3K9me3 at genomic regions including retrotransposons, rDNA, and zinc finger genes [56,57,58]. Intriguingly, ATRX maintenance of heterochromatin at certain loci has been shown to be independent of histone H3.3 deposition with DAXX, and therefore it potentially does not require ATP-dependent chromatin remodeling for this function [56,57]. It is intriguing to consider the possibility that ATRXt could thus function on its own to maintain heterochromatin at a subset of H3K9me3 loci. A recent study has demonstrated that the ADD domain of FL-ATRX, retained in ATRXt, can act as a versatile recognition module of chromatin regulators. In addition to recognizing H3K9me3, the ADD domain can also directly recognize histone macroH2A and CHD4, a crucial component of the chromatin-regulating NuRD complex [58,59]. It will be interesting to address in future work if the ADD domain of ATRXt, independent of ATPase function, could serve as a hub for recruitment of chromatin-regulating proteins to establish and maintain heterochromatin at H3K9me3-enriched regions. Our finding that ATRXt represses rRNA transcription suggests that ATRXt could play a role in the establishment and maintenance of heterochromatin that was previously attributed to FL-ATRX. Future work will more directly examine if ATRXt can promote H3K9me3 heterochromatin formation.

Although we did not look at telomeric loci specifically, the localization of ATRXt to H3K9me3-decorated heterochromatin and rDNA suggests ATRXt can likely occupy telomeric regions, as these regions are enriched in H3K9me3, and FL-ATRX is known to maintain heterochromatin at telomeres [18]. Additionally, it has recently been shown that ATRX binds to the MRE11-RAD50-NBS1 (MRN) complex through an SDT-like motif located at amino acids 750–752 [60]. Given that this motif is also present in ATRXt, and that FL-ATRX has been shown to sequester the MRN complex and thereby attenuate its replication fork degradation, it is interesting to speculate whether ATRXt could achieve the same effect. Nevertheless, we did not test for this in our study and thus this remains an interesting avenue to pursue for future work on ATRXt.

In summary, we provide the first evidence for specific biochemical functions of the short isoform of the ATRX protein, ATRXt. We demonstrate that some of the activities and protein interactions attributed to FL-ATRX can also be performed by ATRXt, drawing into question why two isoforms are necessary and why they have been conserved during evolution. One distinction that is clear is that ATRXt represents a small fraction of the total ATRX protein (5–20%), which may prove challenging when clarifying a specific role for this isoform. Regardless, the U2OS cell lines developed in this study provide an opportunity to unlock redundant functions from isoform-specific functions, beginning with the genomic occupancy mapping of both isoforms. Overall, our data provide strong evidence of previously unappreciated biochemical functions for ATRXt that highlight a need to examine specific roles of FL-ATRX and ATRXt proteins in future studies, particularly since one protein has remodeling activity while the other one does not.

## 4. Materials and Methods

### 4.1. Antibodies

The following primary antibodies were used: ATRX 39F, WB: 1:1000, IF: 1:100, ATRX H-300 (Santa-Cruz, Dallas, TX, USA), IF: 1:500, IP: 3 µg/mL lysate, M2 FLAG (Millipore Sigma, Oakville, ON, Canada) WB: 1:1000, IF 1:500, IP: 3 µg/mL lysate, Vinculin (Abcam, Toronto, ON, Canada) ab91459, WB: 1:5000, H3K9me3 (Abcam ab8898, IF: 1:500), PML (Abcam ab179466, IF: 1:500), DAXX (Thermo Scientific, Ottawa, ON, Canada) MA1-19731, WB: 1:1000, HP1α (Abcam ab77256, WB: 1:500, IF: 1:50), UBF (Santa Cruz sc-13125, IF 1:100), Fibrillarin (Abcam ab4566, IF: 1:500), MAP2 (Sigma ab5622, IF: 1:1000), TUJ1 (BioLegend, San Diego, CA, USA) 801213, IF: 1:1000), MHC (R&D Systems, Burlington, ON, Canada) MAB4470, IF: 1:25, EZH2 (Cell Signaling, Whitby, ON, Canada) 5246, IF: 1:100), H3K27me3 (Invitrogen, Ottawa, ON, Canada PA5-31817, IF: 1:500), Nucleolin (Sigma 05-565, IF: 1:100).

### 4.2. Cell Lines and Cell Culture

U2OS (ATCC HTB-96), HEK 293 (ATCC CRL-1573), SH-SY5Y (ATCC CRL-2266), HeLa (ATCC CRM-CCL-2) and C2C12 (ATCC CRL-1772) cells were cultured in DMEM (Gibco) supplemented with 10% fetal bovine serum (FBS) (Sigma F1051) and 1% penicillin/streptomycin (Thermo 15140122). LUHMES (ATCC CRL-2927) cells were cultured in DMEM/F12 (Gibco) supplemented with 1X N2 (Thermo Scientific 17502-048) and 40 ng/mL bFGF (Thermo Scientific 13256029). The differentiation media for each respective cell line were as follows: for SH-SY5Y, DMEM supplemented with 3% FBS and 10μM retinoic acid; for C2C12, DMEM supplemented with 1% horse serum (HS); for LUHMES cells, DMEM/F12 supplemented with 1X N2 and 1 μg/mL of doxycycline.

U2OS pCMV-Tet3G cells were generated by transfection of pCMV-Tet3G (Clontech, San Jose, CA, USA) 631399 followed by selection after 96 h with 1 mg/mL of G418. Transfections were performed with Lipofectamine 2000 (Fisher, Ottawa, ON, Canada) 11-688-019. Stable clones were maintained with 700 μg/mL of G418. To generate double-stable doxycycline-inducible cell lines, U2OS pCMV-Tet3G cells were transfected either with pTRE3G-BI-ZsGreen, pTRE3G-BI-ATRXt-ZsGreen, or pTRE3G-BI-FL-ATRX-ZsGreen, followed by selection after 96 h with 1 μg/mL of puromycin. Stable clones were maintained in 0.5 μg/mL of puromycin. All cell lines were regularly tested for mycoplasma.

U2OS cells constitutively expressing ATRXt were generated by transfecting U2OS cells with pBRIT-LoxP-ATRXt-NTAP, followed by selection with 1 μg/mL of puromycin after 48 h. Stable clones were maintained in 0.5 μg/mL of puromycin.

### 4.3. Nuclease Treatments

U2OS^ATRXt^ or U2OS^FL−ATRX^ cells were seeded on glass coverslips with 1 μg/mL doxycycline added to growth media for 24 h prior to nuclease treatments. Cells were first permeabilized for 5 min with ASE buffer (20 mM Tris pH 7.5, 5 mM MgCl_2_, 0.5 mM EGTA) with 0.1% triton X-100 added just prior to use. Cells were then either treated with DNAse (100 μg/mL) or RNAse (200 μg/mL) dissolved in PBS for 20 min at 37 °C (or only PBS in the case of mock treated cells), followed by fixation in 4% paraformaldehyde (PFA) for 10 min. Samples were then processed for immunofluorescence as described.

### 4.4. Immunoprecipitation

Cells were transfected 48 h prior to harvesting for IP. Cells were rinsed 1X with PBS and lysed on ice for 10 min with IP buffer (20 mM Tris pH 8, 420 mM NaCl, 10% glycerol, 2% NP-40, 2 mM EDTA) with protease inhibitors added just prior to use. DNAse was added (10 μg/mL), and the samples were incubated at 37 °C for 5 min. Samples were centrifuged at 4 °C for 5 min at 13,000 rpm. The supernatant was transferred to a new tube and assayed for protein concentration using Bradford reagent. A total of 20 μL of protein A magnetic beads (Thermo Scientific 88845) were rinsed 1X in IP buffer and resuspended in 200 μL of PBST (PBS with 0.1% Tween-20), and 3 μg of antibody or species matched IgG was added to the bead slurry, followed by incubation on a nutating mixer for 1 h at 4 °C. The beads were then washed once more in IP buffer and then mixed with cell lysate totaling 1 mg of protein and incubated overnight on a nutating mixer at 4 °C. The following morning, the supernatant was removed, and the beads were washed 3X for 5 min in IP buffer with 0.1% Tween-20, followed by one additional wash in IP buffer without Tween. Proteins were eluted by adding 10 μL of 1 M 2-mercaptoethanol and 10 μL of 4X LDS sample buffer (Invitrogen NP0007) followed by boiling for 5 min at 95 °C.

### 4.5. Western Blotting

Protein lysates were diluted in 4X LDS sample buffer supplemented with 15% *v*/*v* of 2-mercaptoethanol prior to boiling for 5 min at 95 °C. A total of 20 μg of lysate was loaded onto 6, 8 or 10% Tris-Glycine gel and run at 175 V for 45 min in 1X running buffer (25 mM Tris, 192 mM glycine, 0.1% SDS). Proteins were then transferred onto activated PVDF membranes for 2–3 h at 200 mA in 1X transfer buffer (200 mM glycine, 25 mM Tris, 20% methanol) using a wet transfer system. Membranes were then blocked in 5% milk in 1X TBST (20 mM Tris, 150 mM NaCl, 0.1% Tween-20) for 1 h, followed by incubation with primary antibody diluted in 1X TBST overnight at 4 °C on a nutating mixer.

### 4.6. Immunofluorescence

Cells were seeded on coverslips and fixed in 4% PFA for 10 min. Cells were then washed 2X with 1X PBS and permeabilized with 0.5% NP-40 in IF solution (0.5% BSA in PBS) for 10 min. Cells were quenched with 50 mM ammonium chloride in PBS for 10 min and then blocked for 10 min with 10% horse serum in IF solution. The coverslips were incubated in primary antibody diluted in IF solution at room temperature for 1 h in a humidity chamber, washed 3X for 5 min in IF solution and incubated in fluorophore-conjugated secondary antibody diluted in IF solution for 1 h in a humidity chamber at room temperature. Coverslips were washed again 3X for 5 min in IF solution and incubated with DAPI (1 μg/mL) for 5 min and pyronin Y (Sigma 83200) at 0.75 μM for 5 min if required. Coverslips were then rinsed with IF solution and mounted onto slides with DAKO fluorescence mounting medium (Sigma M1289).

### 4.7. Cloning of Plasmid Constructs

For use with transient transfections, ATRXt cDNA was generated by HiFi Assembly (NEB, Whitby, ON, Canada) E5520 of PCR-amplified human FL-ATRX exons 1–10, peGFP-C3, and human ATRX exon 10a, which was synthesized commercially (GeneWiz, Waltham, MA, USA). Using this ATRXt-peGFP construct, ATRXt cDNA was then PCR-amplified and directionally cloned into the pBRIT-LoxP-NTAP retroviral vector. ATRX exon 10a was PCR-amplified from ATRXt-peGFP and directionally cloned into peGFP-C3. To generate constructs for use with the Tet-On system, FL-ATRX and ATRXt cDNA was PCR-amplified and cloned into linearized pTRE3G-BI-ZsGreen using the In-Fusion cloning kit (Clontech).

### 4.8. RNA Extraction and RT-qPCR

Cells were grown to 80% confluence on a 6-well plate prior to RNA extraction. Cells were rinsed with PBS followed by addition of 1 mL of Trizol. The lysate was transferred to a tube and incubated for 5 min followed by addition of 250 μL of chloroform. The lysates were immediately vigorously vortexed for 15 s and further incubated for 5 min. Samples were then centrifuged for 10 min at 14,000 rpm and the aqueous phase pipetted into new tubes and immediately mixed with 550 μL of isopropanol. The mixture was incubated for 5 min and then centrifuged for 30 min at 14,500 rpm at 4 °C. The RNA pellet was washed 2X with 75% ethanol, then dried and resuspended in 20 μL of HPLC grade water. A total of 2 μg of RNA was treated with DNA-free DNA removal kit (Invitrogen 1906) and used for reverse transcription (RT) using a RevertAid Reverse Transcription Kit (Thermo K1691) to generate cDNA. RT samples were prepared for qPCR using SYBR green (FroggaBio, Concord, ON, Canada) BIO-94020 and qPCR reactions were performed on an AriaMx real-time PCR system (Agilent, Santa Clara, CA, USA). Fold changes were calculated using the ΔΔCt method using POLR2A as an internal control.

### 4.9. ChIP

U2OS^ATRXt^ cells were seeded with or without 1 μg/mL doxycycline added to growth media for 24 h prior to the ChIP. Confluent cells grown on a 10 cm dish were crosslinked with 1% formaldehyde for 10 min, followed by quenching with 1.25 M glycine and then cell lysis with SDS lysis buffer (2% SDS, 10 mM EDTA, 50 mM Tris pH 8.1), with protease inhibitors added just prior to use. Lysate was passed through a 25 G needle followed by sonication on 10% output for 4 pulses of 10 s each. Samples were centrifuged for 5 min at 13,000 rpm at 4 °C. The supernatant was transferred to a new tube and assayed for protein concentration using Bradford reagent. A total of 70 μg of chromatin was diluted in a mixture of 900 μL of ChIP dilution buffer (167 mM NaCl, 16.7 mM Tris pH 8.1, 1.1% Triton X-100, 1.2 mM EDTA, 0.01% SDS) and 100 μL of SDS lysis buffer; 10 μL was removed for a 1% input sample. A total of 50 μL of protein A magnetic beads (Thermo 88845) were rinsed 1X in SDS lysis buffer and resuspended in 200 μL of PBST; then, 4 μg of antibody was added to the bead slurry followed by incubation on a nutating mixer for 1 h at 4 °C. The beads were then washed once more in SDS lysis buffer and then mixed with the diluted chromatin sample and incubated overnight on a nutating mixer at 4 °C. The following morning, the supernatant was removed and the beads were washed with a series of wash buffers in the following order for 3 min each on a nutating mixer at 4 °C: low salt wash buffer (159 mM NaCl, 20 mM Tris pH 8.1, 2 mM EDTA, 1% Triton X-100, 0.1% SDS), twice with high salt wash buffer (300 mM NaCl, 20 mM Tris pH 8.1, 2 mM EDTA, 1% Triton X-100, 0.1% SDS), LiCl wash buffer (250 mM LiCl, 10 mM Tris pH 8.1, 1 mM EDTA, 1% IGEPAL, 1% sodium deoxycholic acid), and twice with TE buffer. DNA was eluted by mixing the beads with 120 μL of elution buffer (1% SDS, 0.1 M NaHCO_3_) and shaking for 15 min, repeated once. A total of 200 μL of elution was mixed with 8 μL of 5 M NaCl and incubated at 65 °C overnight to reverse the crosslinks. Then, 1% input samples were mixed with 190 μL of elution buffer and 8 μL of 5 M NaCl and incubated at 65 °C overnight. The samples were then treated with 1 μL of RNAse A (10 mg/mL) and 1 μg of proteinase K prior to phenol–chloroform extraction and ethanol precipitation of DNA fragments.

### 4.10. Cell Proliferation Assay

U2OS^ATRXt^ or U2OS^FL−ATRX^ cells were seeded to a density of 50,000 cells per well in a 6-well plate in the presence or absence of 1 μg/mL doxycycline, and their growth rate was assessed at the indicated timepoints by cell counting using a Countess 3.

### 4.11. Chemical Treatment Assays

U2OS^ATRXt^ or U2OS^FL−ATRX^ cells were seeded on glass coverslips with 1 μg/mL doxycycline added to growth media for 24 h prior to chemical treatments. The cells were treated with either 0.5 μg/mL Actinomycin D (Sigma) or 25 μg/mL 5,6-dichloro-β-D-ribofuranosylbenzimadole (DRB, Sigma) for 2 h prior to PFA fixation and immunofluorescence as described.

### 4.12. Image Acquisition and Processing

Fluorescent microscope images were captured using an AxioImager M1 (Zeiss, Toronto, ON, Canada) using Zen 3.5 software. For the images collected after chemical treatment assays, fluorescent microscope images were captured using a Zeiss LSM 900 with Airyscan 2 using Zen 3.5 software. Live cell images were captured using an AxioObserver Z1 (Zeiss) with Zen 3.5 software.

Image analysis was performed using Fiji (ImageJ version 1.54p). Fluorescence intensities for nuclease treatments were measured by defining nucleoli as ROIs and measuring the mean fluorescence intensities of each channel within the nucleoli for each condition, followed by plotting in GraphPad PRISM version 8. Dotted line scanning to graph colocalization in Figure 1 was performed using the plot profile function in Fiji. Colocalization analysis for the chemical treatment assays was performed using the Coloc 2 plugin in Fiji. Manders’ colocalization coefficients were calculated and then the values for each condition plotted using GraphPad Prism 8.0.2 (Boston, MA, USA).

## Data Availability

The original contributions presented in this study are included in the article/Supplementary Materials. Further inquiries can be directed to the corresponding author.

## Supplementary Materials

The following supporting information can be downloaded at: https://www.mdpi.com/article/10.3390/ijms27073103/s1.
