# Supplementary material for: The ATRXt Protein Represses rDNA Transcription While Mirroring ATRX Interactions and Heterochromatin Localization
Source: Int J Mol Sci. 2026 Mar 29;27(7):3103. doi: 10.3390/ijms27073103 (PMC13074095; doi:10.3390/ijms27073103)
Supplement: Supplementary file 1 [file ijms-27-03103-s001.zip › ijms-4215369-supplementary.pdf]

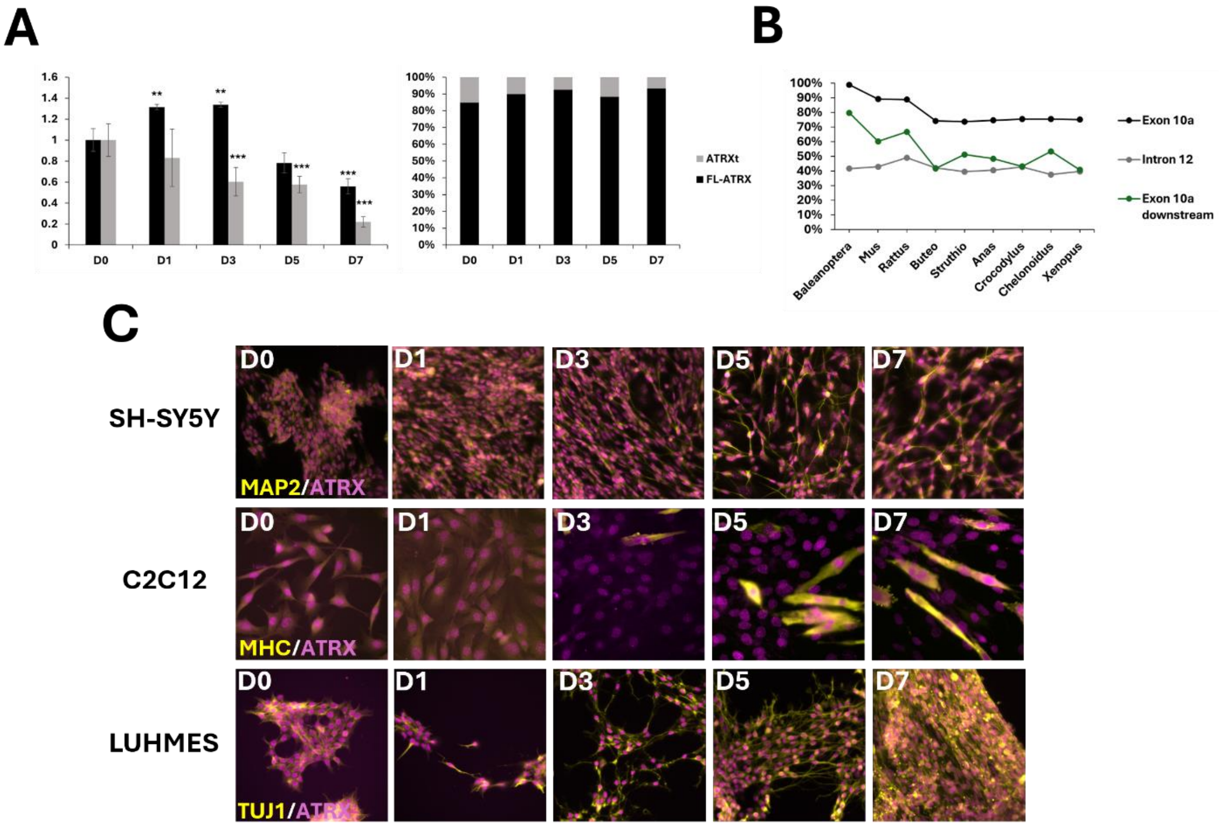

Supplementary Figure S1. (**A**) Left, the levels of ATRXt and FL-ATRX mRNA measured in differentiating C2C12 cells. The x-axis indicates the timepoint in days following initiation of differentiation, with D0 being undifferentiated, proliferating cells. The y-axis indicates the fold change in mRNA levels relative to proliferating cells. Right, the proportion of ATRX transcript made up by FL-ATRX and ATRXt at the same timepoints as in the graph on the left. (**B**) The homology of ATRXt exon 10a nucleotide sequence compared to other ATRX nucleotide sequences. The genomic sequence of exon 10a exhibits a substantially higher degree of homology in various species versus the region immediately downstream, or versus the intronic sequence immediately downstream of exon 12. (**C**) Immunofluorescence images of SH-SY5Y, LUHMES, and C2C12 cells at specified timepoints throughout differentiation showing changes in cell morphology and ATRX expression. Scale bar = 50μm.

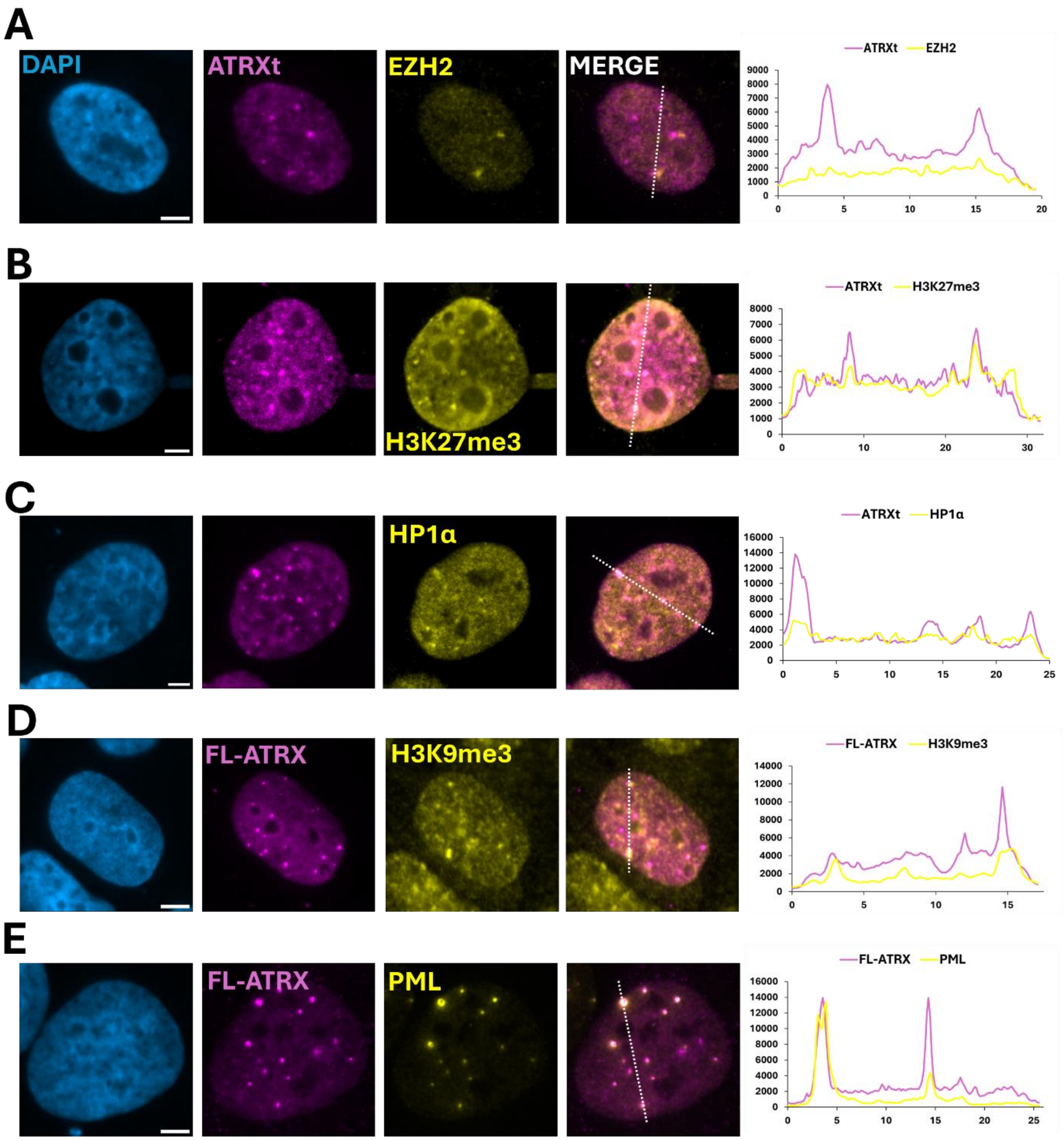

Figure S2. ATRXt & FL-ATRX colocalize with numerous heterochromatin factors. Comparison of colocalization of overexpressed ATRXt in U2OS cells with EZH2 (**A**), H3K27me3 (**B**), and HP1α (**C**). Overexpressed FL-ATRX colocalization with H3K9me3 and PML is shown in (**D**) and (**E**), respectively. Dotted line scanning is shown on the right for each panel to indicate colocalization.

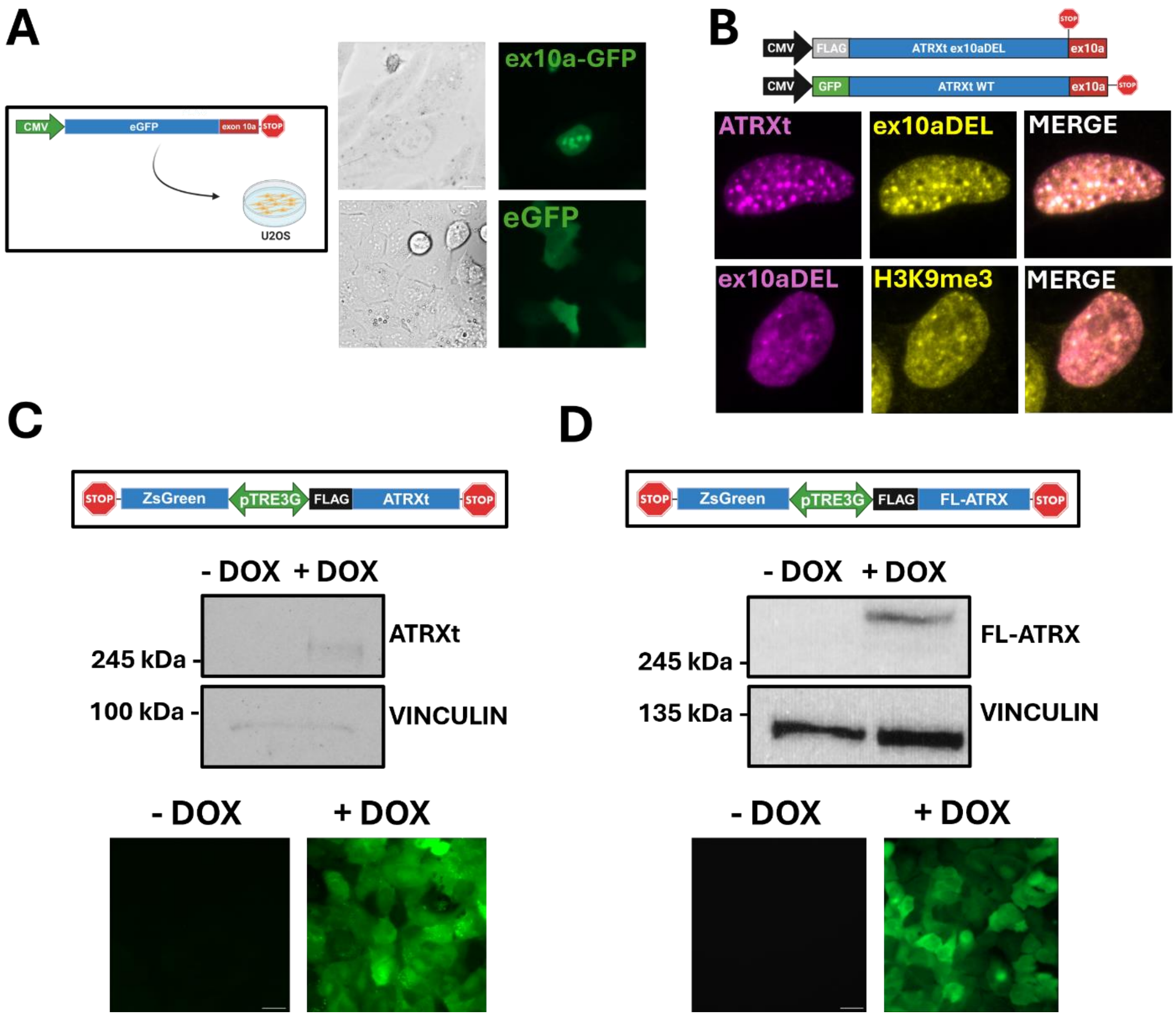

Supplementary Figure S3. (**A**) Top, schematic showing eGFP-ATRXt exon10a construct. Bottom, comparison of the cellular localization of eGFP-exon10a construct (top) versus only eGFP (bottom). (**B**) Top, schematic of ATRXt constructs used to express ATRXt WT or ATRXt with exon 10a deleted; middle, co-staining of ATRXt WT (tagged with GFP) and ATRXt with exon 10a deleted (tagged with FLAG) after co-transfection in U2OS cells; Bottom, exogenously expressed ATRXt with exon 10a deleted co-stained with H3K9me3 in U2OS cells (**C**) Top, schematic of the expression construct used for doxycycline-inducible expression of ATRXt and ZsGreen in U2OS cells; middle, western blot showing ATRXt expression in U2OS^{ATRXt} cells after 1 day of doxycycline treatment; bottom, ZsGreen expression in U2OS^{ATRXt} cells after 1 day of doxycycline treatment. (**D**) Top, schematic of the expression construct used for doxycycline-inducible expression of FL-ATRX and ZsGreen in U2OS cells; middle, western blot showing FL-ATRX expression in U2OS$^{ATRX\text{-}FL}$ cells after 1

day of doxycycline treatment; bottom, ZsGreen expression in U2OS$^{ATRX\text{-}FL}$ cells after 1 day of doxycycline treatment. Scale bar = 5μm.

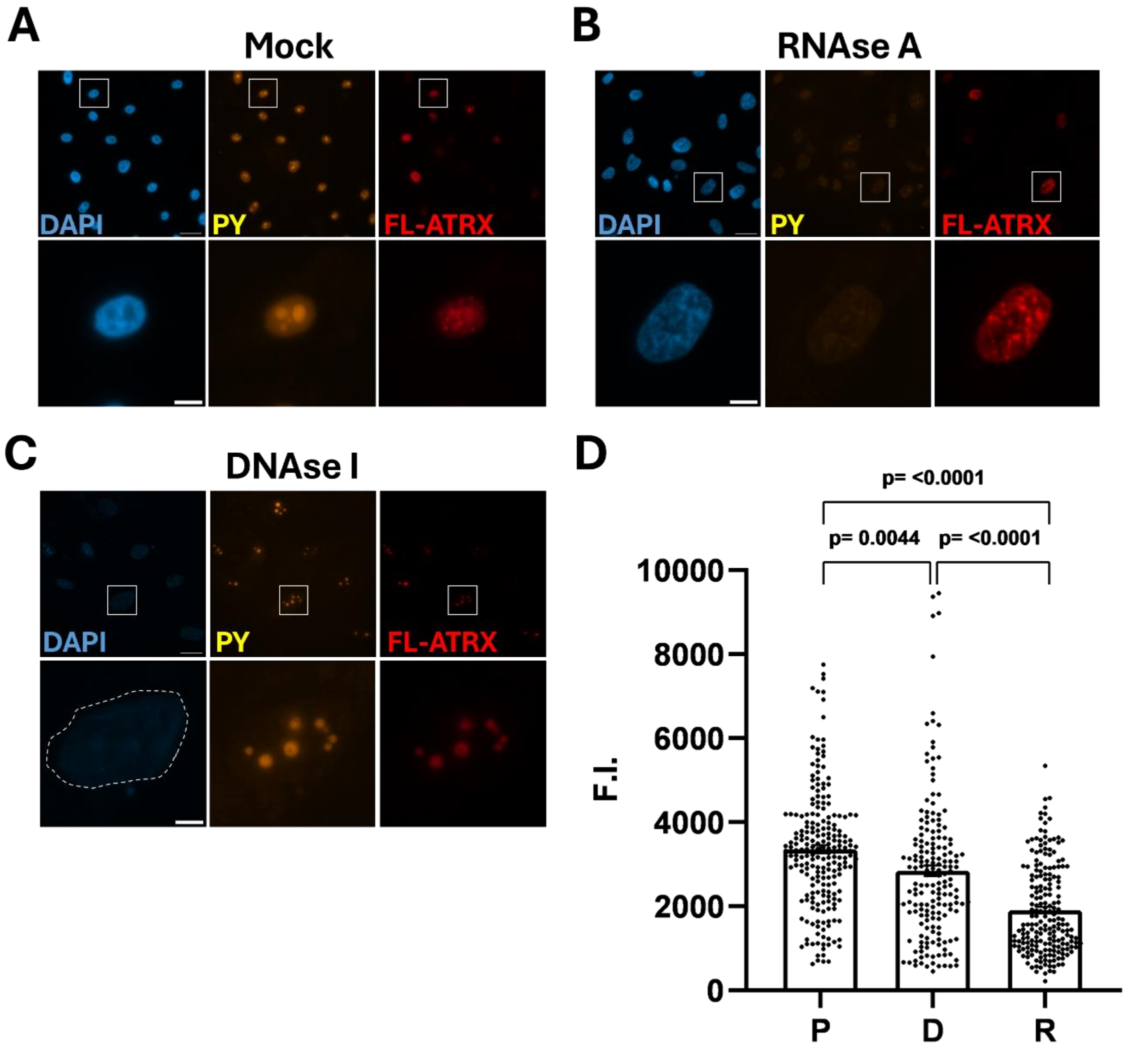

Supplementary Figure S4. (**A**) U2OS$^{ATRX\text{-}FL}$ cells were either permeabilized or treated with (**B**) RNAse A (200μg/mL) or (**C**) DNAse I (100μg/mL) 24 hours after induction of ATRXt expression. (**D**) Quantification of FL-ATRX nucleolar intensity after permeabilization (P), DNAse I (D) or RNAse A (R) treatment. Y axis = fluorescence intensity (arbitrary units). # of nucleoli counted per condition: P = 216, D = 180, R = 199. Average # of nucleoli per cell = 3.82, n=151. Scale bar = 20μm, inset = 5μm.

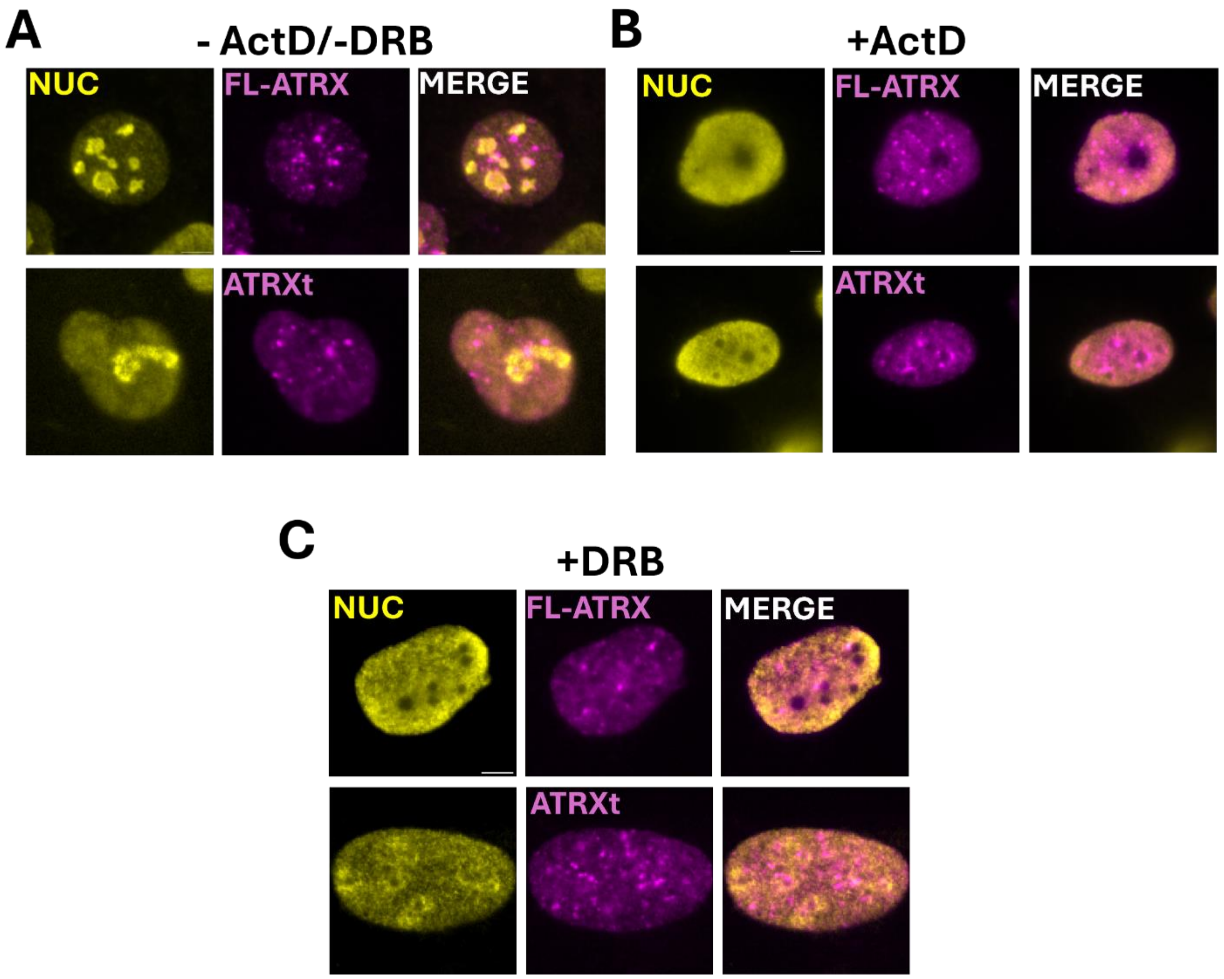

Supplementary Figure S5. U2OS^{ATRXt} or U2OS$^{ATRX\text{-}FL}$ were induced for ATRXt or FL-ATRX expression respectively for 24 hours and were either untreated (**A**) or subsequently treated with 0.5 µg/ml Actinomycin D for 2 hours (**B**) or with 25 µg/ml 5,6-dichloro-*β*-D-ribofuranosylbenzimadole for 2 hours (**C**) and co-stained for ATRXt or FL-ATRX with nucleolin. Scale bar = 5µm.

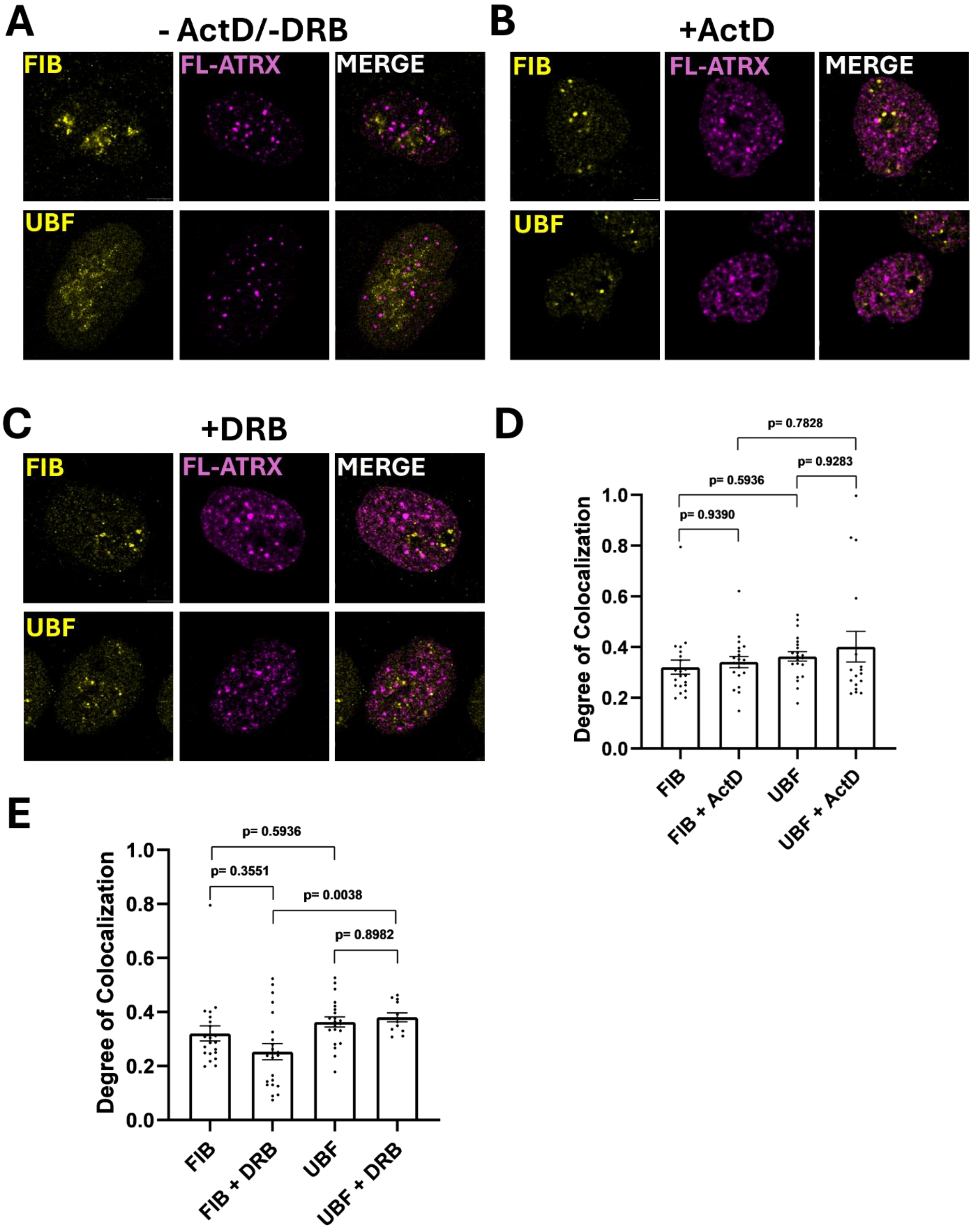
A
- ActD/-DRB
FIB
FL-ATRX
MERGE
UBF
B
+ActD
FIB
FL-ATRX
MERGE
UBF
C
+DRB
FIB
FL-ATRX
MERGE
UBF
D
Degree of Colocalization
p= 0.7828
p= 0.9283
p= 0.5936
p= 0.9390
FIB
FIB + ActD
UBF
UBF + ActD
E
Degree of Colocalization
p= 0.5936
p= 0.3551
p= 0.0038
p= 0.8982
FIB
FIB + DRB
UBF
UBF + DRB

Supplementary Figure S6. FL-ATRX colocalization with nucleolar sub-compartments. U2OS$^{ATRX\text{-}FL}$ cells were treated with doxycycline for 24 hours and subsequently co-stained for ATRXt with UBF or fibrillarin (FIB), markers of the FC and DFC, respectively. Cells were either untreated (**A**), treated with 0.5 μg/ml Actinomycin D for 2 hours (**B**) or treated with 25 μg/ml 5,6-dichloro-*β*-D-ribofuranosylbenzimadole for 2 hours (**C**). (**D**) The degree of colocalization (Manders' coefficient) of FL-ATRX with UBF and fibrillarin before and after Actinomycin D treatment. (**E**) The degree of colocalization (Manders' coefficient) of FL-ATRX with UBF and fibrillarin before and after 5,6-dichloro-*β*-D-ribofuranosylbenzimadole treatment. # of nuclei counted per condition: FIB = 21, UBF = 21, FIB w/ ActD = 20, UBF w/ ActD = 17, FIB w/ DRB = 22, UBF w/ DRB = 12.

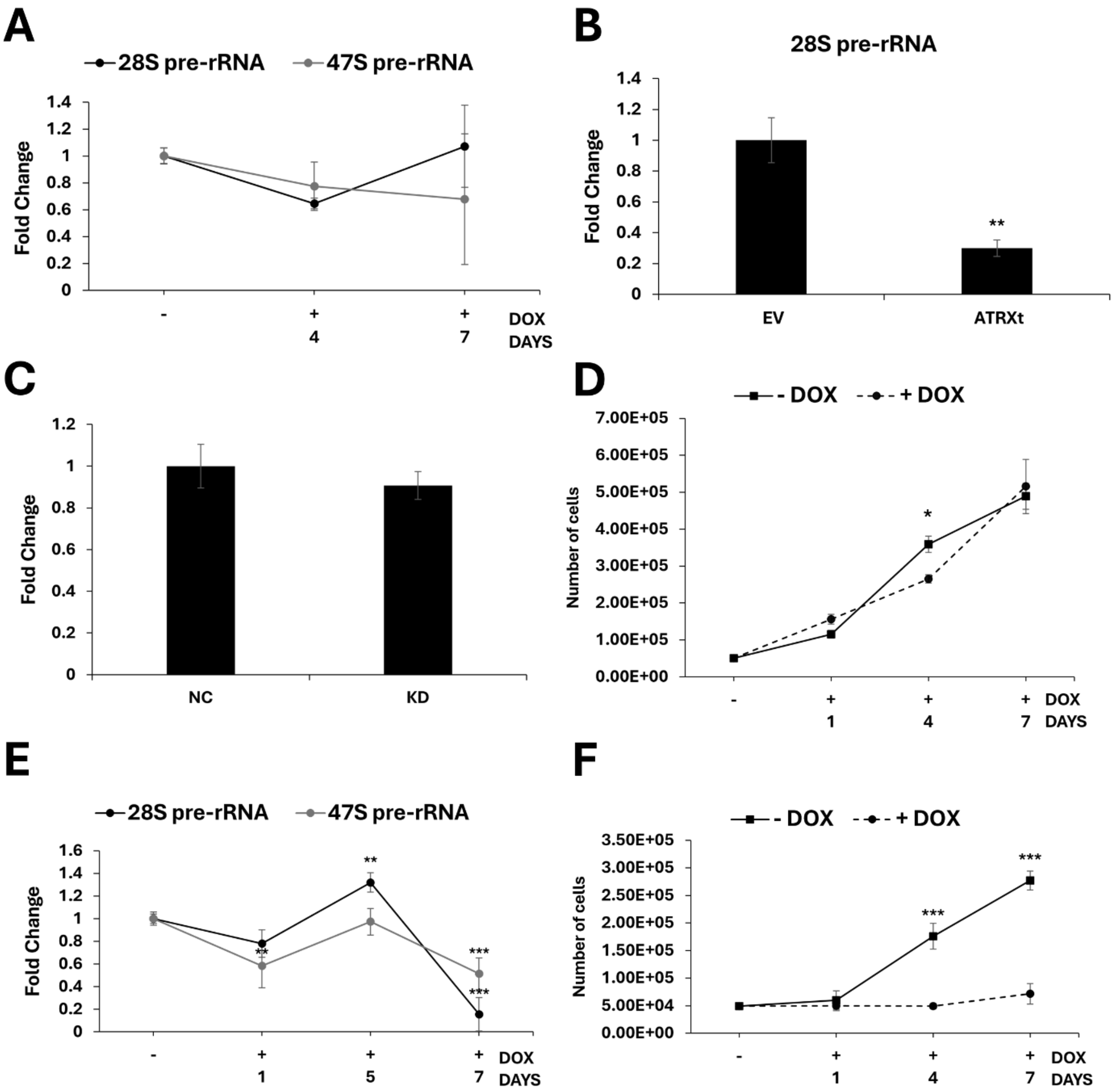

A
28S pre-rRNA
47S pre-rRNA
Fold Change
DOX
DAYS
B
28S pre-rRNA
Fold Change
EV
ATRXt
**
C
Fold Change
NC
KD
D
- DOX
+ DOX
Number of cells
*
DOX
DAYS
E
28S pre-rRNA
47S pre-rRNA
Fold Change
**
**
***
***
DOX
DAYS
F
- DOX
+ DOX
Number of cells
***
***
***
DOX
DAYS

Supplementary Figure S7. (**A**) Fold change of 47S and 28S rRNA levels in U2OS^{ZsGreen} cells at selected timepoints up to 7 days after doxycycline-treatment relative to rRNA levels in cells with no doxycycline added. (**B**) 28S pre-rRNA levels in U2OS cells which were puromycin-selected to constitutively express ATRXt measured relative to U2OS cells which were puromycin-selected to express only empty vector. (**C**) Fold change of FL-ATRX transcript in HeLa cells 24 hours after siRNA knockdown (KD) of ATRXt, measured relative to cells treated with non-targeting siRNA (NC). (**D**) Number of U2OS^{ZsGreen} cells 1, 4 and 7 days after plating, with and without doxycycline treatment. 50000 cells were plated on the first day. (**E**) Fold change of 47S and 28S rRNA levels in U2OS$^{ATRX\text{-}FL}$ cells at selected timepoints up to 7 days after doxycycline-treatment relative to rRNA levels in cells with no doxycycline added. (**F**) Number of U2OS$^{ATRX\text{-}FL}$ cells 1, 4 and 7 days after plating, with and without doxycycline treatment. 50000 cells were plated on the first day. n = 3 for all experiments, *=p<0.05, **=p<0.01, ***=p<0.001.
